# A novel proceduralized donor liver back-table preparation technique minimizes hemorrhage following liver implantation in orthotropic liver transplantation

**DOI:** 10.3389/fsurg.2024.1356142

**Published:** 2024-12-12

**Authors:** Rui Tang, Guangdong Wu, Xuan Tong, Lihan Yu, Ang Li, Jingyi Lin, Guangxun Xu, Qian Lu

**Affiliations:** Hepatopancreatobiliary Center, Beijing Tsinghua Changgung Hospital, School of Clinical Medicine, Institute for Precision Medicine, Tsinghua University, Beijing, China

**Keywords:** back-table procedure, hepatic artery variation, orthotropic liver transplantation, procedural donor liver preparation, extracorporeal donor liver preparation

## Abstract

**Background:**

Intraoperative hemorrhage is one of the major complications of orthotopic liver transplantation (OLT_X_) and is mainly caused by technical difficulties of the surgical procedure besides primary liver diseases. The present study aimed to evaluate the feasibility and clinical effects of a novel proceduralized donor liver back-table preparation (DLBTP) technique for use in OLT_X_.

**Methods:**

This retrospective study was conducted between January 2018 and June 2020 based on patients who had undergone OLT_X_. All livers transplanted using the reported back-table procedures were defined as the control group A (*n* = 43), while those prepared using our new procedure as the experimental group B (*n* = 160). The first-hand surgical experience of transplant surgeons was surveyed in a *post hoc* comparative analysis.

**Results:**

DLBTP time was significantly longer and the probability of low-quality hepatic artery skeletonization was lower in group B compared to group A patients. Compared to group A, the time for hemorrhage control was shorter [*P* < 0.05, 0.3 h (range, 0.17–0.58 h)], and the degree of blood loss was less [*P* < 0.05, 60 ml (range, 30–240 ml)] in group B. Major bleeding sites were soft tissue of the hepatic hilum and the wall of the inferior vena cava. Due to trimmed soft tissue in the first porta hepatis region, there was less blood oozing, making it easier to stem the bleeding and construct anastomosis.

**Conclusion:**

Although the procedural DLBTP for OLT_X_ was time-consuming, the new procedure significantly reduced the degree of hemorrhage and the time required to control bleeding.

## 1 Introduction

During the past three decades, advances in surgical techniques have improved the clinical outcomes of orthotopic liver transplantation (OLT_X_). With an improved anatomical knowledge of variations in the hepatic artery, the development of surgical techniques of vascular anastomosis, the availability of vascular grafts and other innovative technologies of revascularization, the 1-year liver graft loss due to arterial complications has significantly dropped from 10% to 2% (1). One of the underlying reasons for this drop has been the improvement of back-table preparation of the procured donor livers (2).

As the bridge step from the procurement of donor livers to their implantation, back-table preparation, which includes a series of extracorporeal dissections and skeletonization of the hepatic vasculature and biliary ducts of donor livers, has been considered one of the impact factors to prolong the cold ischemia time, subsequently increasing the chance of graft failure (3). However, previous studies have not provided a detailed description of the back-table preparation procedures. Back-table preparation of donor liver for OLT_X_ can be proceduralized as a stepwise operation, which involves the removal of the perihepatic ligament, trimming of connective tissue around the hepatic hilum, and skeletonization of the retrohepatic inferior vena cava (IVC), portal vein (PV), and hepatic artery, and separation of the bile duct by careful dissection. This improved preparation not only benefits the *post hoc* transplantation procedure but also is crucial for early patient recovery from surgery (4).

Two big challenges face surgeons carrying out the back-table preparation of donor livers. On the one hand, it has been postulated that because a hemorrhage cannot be visualized during back-table preparation, a small blood vessel break may go unnoticed, resulting in bleeding after the PV is opened. On the other hand, during graft preparation, the misrecognition of a variant hepatic artery may cause hepatic artery thrombosis (HAT) and injury, a serious complication of OLT_X_ (5). The present study aims to evaluate the feasibility and clinical effects of a novel proceduralized donor liver back-table preparation (DLBTP) for use in OLT_X_.

## 2 Methods

### 2.1 Ethical approval

This study was based on the Declaration of Helsinki principles and was approved by the ethics committee of Beijing Tsinghua Changgung Hospital (approval number: 21411-6-01). Written informed consent was obtained from all enrolled patients.

### 2.2 Inclusion and exclusion criteria

A retrospective cohort study was conducted based on patients who underwent DLBTP procedures for OLT_X_ and its clinical effectiveness at a university hospital between January 2018 and June 2020. The criteria for inclusion in the study were as follows: (1) the donor livers were back-table prepared during the study period, and (2) the prepared donor livers were implanted during OLT_X_. The exclusion criteria were as follows: (1) an injury was found along the aberrant hepatic artery during the donor liver harvesting procedure; (2) the donor age was <18 years; (3) the donor liver could not be used in OLT_X_ or changed to partial liver transplantation such as split liver or reduced-size liver transplantations; and (4) incomplete clinical data.

### 2.3 Groupings

Surgeons performing the implantations all had considerable experience, having independently completed over 100 liver transplant surgeries, earning industry recognition. Group A was designated as the control group and included patients who had other back-table procedures carried out by two transplant surgeons, who had completed more than 50 back-table procedures. Patients with the back-table procedure described in the present study performed by another surgeon, who had completed 20 back-table procedures in the past, were designated as group B (the experimental group).

### 2.4 Procedural back-table donor liver preparation procedure

The back-table procedure on a donor liver was initiated by an overall examination of the liver texture, verifying whether any kind of injury existed, assessing the intactness of the vasculature including the vena cava, hepatogastric ligament, hepatoduodenal ligament, as well as visualizing the superior mesenteric vein (SMV) catheterization and the resection margin of the pancreatic head. Afterward, the back-table donor liver preparation procedure continued in a stepwise fashion as follows (Supplementary Video S1).

#### 2.4.1 Step 1: preparation of the IVC

The first step was the preparation of the IVC. The vascular wall of the infra-hepatic IVC was pulled up to dissect the IVC free from its posterior attachment. Next, the right adrenal gland was separated, the right adrenal vein sutured, and the right adrenal gland resected. The diaphragm was severed from the tissues of the posterior wall of the IVC, and the blood vessels and lymphatic vessels in these tissues were ligated. To improve the exposure of the retrohepatic IVC, the right deltoid and coronary ligaments were cut open to liberate the diaphragm from the liver to the right edge of the IVC. If veins were found to branch from the diaphragm, they were suture-ligated.

**Figure 1:**
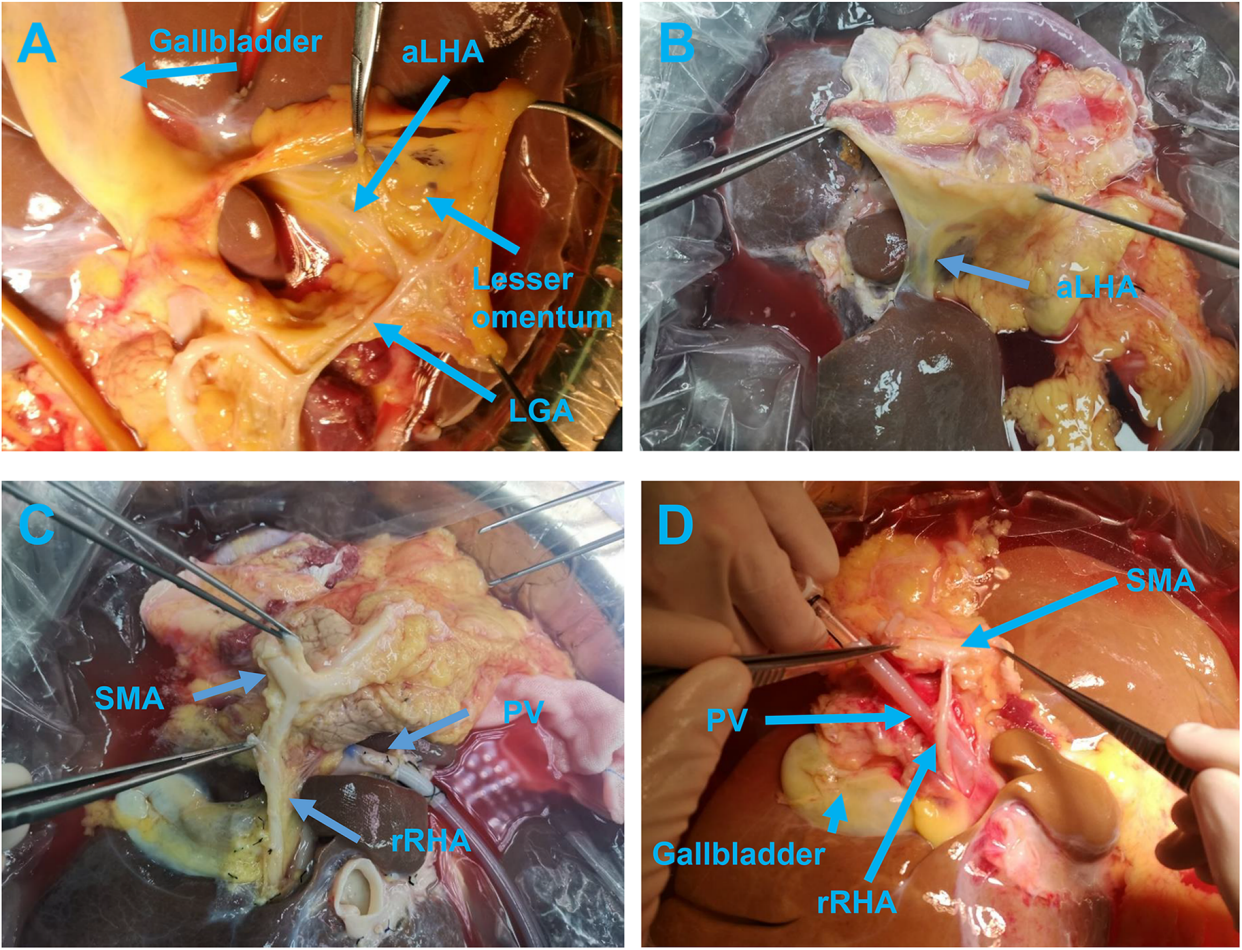
The anterior view **(A)** and posterior view **(B)** of the aLHA branching out from the LGA and running through the hepatogastric ligament. Initial dissection of the rRHA arising from the SMA **(C)** and after exposure running posterior to the PV **(D)**. aLHA, accessory left hepatic artery; LGA, left gastric artery; PV, portal vein; rRHA, replaced right hepatic artery; SMA, superior mesenteric artery.

Moreover, the wall of the supra-hepatic IVC was gently held up to dissect and free the upper supra- and post-hepatic IVC from their posterior attachments, revealing the entire posterior wall of the IVC and further freeing the supra-hepatic IVC from both sides. The sickle, left triangular, and coronary ligaments were cut, and the diaphragmatic membrane was incised open in the midline of the anterior wall of the supra-hepatic IVC and, then, separated on both sides. During this dissection sequence, the diaphragmatic vein was suture-ligated. Once the IVC preparation was completed, the hepatogastric ligament was transected. In this process, the surgeon ensured that the transaction was not too close to the liver and carefully checked whether an aberrant left accessory artery existed to avoid any damage to the blood vessels of the left liver (Figures 1A,B).

#### 2.4.2 Step 2: preparation of the PV

The fibrous membrane covering the posterior wall of the PV was cut open and separated from the liver distally and proximally to expose the wall, and the peripheral nerves and lymphatic vessels around the PV were isolated and ligated near the liver. In this region, the surgeon's attention was focused on determining whether a hepatic artery was present around the PV to avoid accidental damage caused by large-scale trimming of the surrounding tissue. The posterior walls of the PV and SMV were continually dissected from their attachments up to the distal hepatic side. Once the dissection reached the uncinate process of the pancreas, the aberrant hepatic arteries were the focal point for the preparation. Usually, the aberrant arteries, arising from the superior mesenteric artery (SMA), celiac trunk (CT), common hepatic artery (CHA), or splenic artery (SpA), can be frequently identified in this region and are commonly found across the posterior walls of the PV and SMV. If a variant hepatic artery was found, it was dissected free along the course of the variant artery toward the abdominal aorta. If the aberrant hepatic artery arose from the SMA, the SMA segment was retained with the artery (Figures 1C,D).

**Figure 2:**
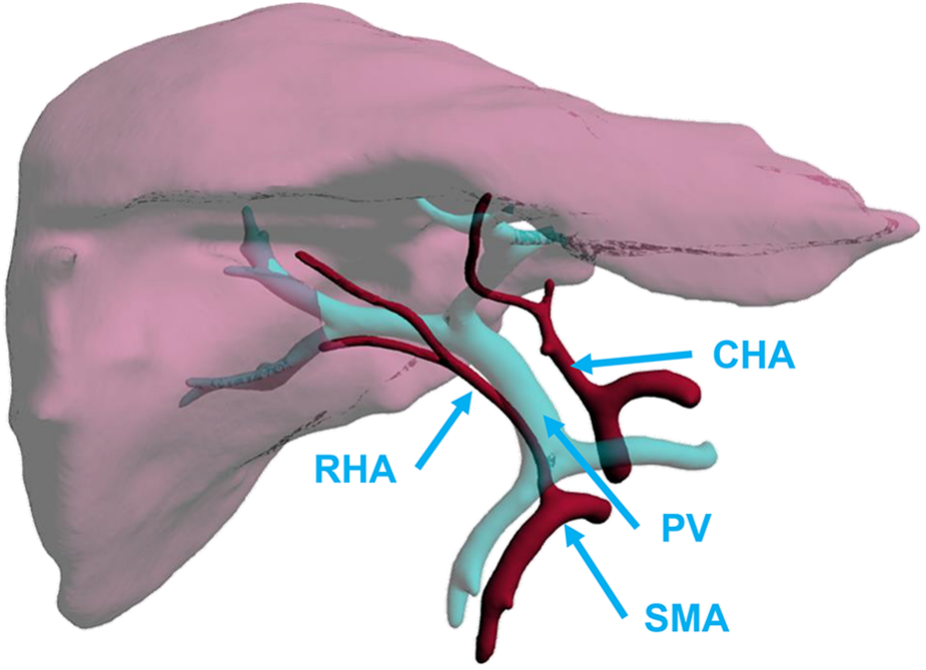
Aberrant RHA arising from SMAs. CHA, common hepatic artery; PV, portal vein; RHA, right hepatic artery; SMA, superior mesenteric artery.

The SMA was gently dragged to the right hepatic side after the abdominal aorta was transected and dissected along the left edge of the SMA toward the SMV. This dissection method can bypass the aberrant hepatic artery that arises from the SMA and goes across and behind the PV, without the requirement to spend time ensuring that the variant hepatic artery presents behind the PV and near the uncinate process of the pancreas (Figure 2). If the variant hepatic artery behind the PV does not originate from the SMA, then to avoid injury, excessive separation was not carried out. Here, the blood vessel is detoured to continue the dissection of the SMV to the foot side until the cannula. Then, the surrounding tissues of the SMV were separated and the SMV, as well as the PV, were completely dissected free from their attachments toward the liver in a reverse direction. During dissection of the SMV and PV, all the branches flowing into the SMV and PV including the splenic vein and pancreatic reflux vein were suture-ligated, completing the preparation of the PV. A useful tip is that priority should be given to ligation of the 5 cm segment of the PV near the liver side to prevent shedding and bleeding of surgical knots during the operation.

#### 2.4.3 Step 3: preparation of common bile duct (CBD)

Following the back-table preparation of the PV, the operation turns to the front of the liver to remove a part of the lymph and nervous tissue on the right side of the CBD, but the dissection should not be overly aggressive. Since the lower portion of CBD is wrapped with pancreatic parenchyma, it should be dissected free from the parenchyma. Free CBD can only be 1 cm at most in length after its dissection from the pancreatic segment, and extra attention had to be paid to preserve the surrounding tissues of CBD and to avoid excessive dissection of tissues.

#### 2.4.4 Step 4: preparation of hepatic artery trees

**Figure 3:**
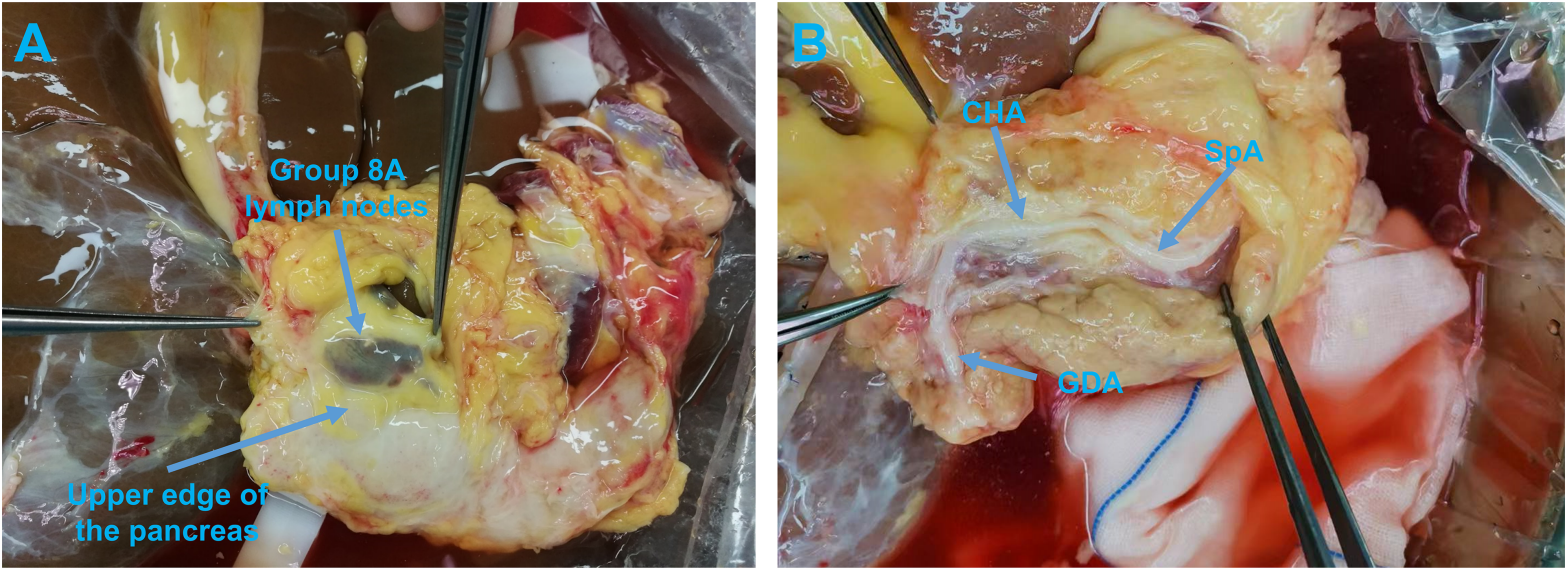
**(A)** Opening the peritoneum between the pancreas and group 8A lymph nodes along the upper edge of the pancreas. **(B)** Exposing the anterior wall of the CHA and GDA. CHA, common hepatic artery; GDA, gastroduodenal artery; SpA, splenic artery.

Now, the hepatic artery can be located by further examining the hepatoduodenal and hepatogastric ligaments. Then, along the upper edge of the pancreas, the peritoneum was opened between the pancreas and group 8A lymph nodes (Figure 3A) to expose the anterior wall of the CHA-gastroduodenal artery (GDA) (Figure 3B). Dissection then moved toward the tail of the pancreas to reveal the anterior wall of the CHA-SpA. Once the skeletonization of SpA, approximately 5 cm from the junction of the CHA and SpA, was completed the SpA was severed and dissected from its attachment in a reverse manner toward the CT. As another branch of the CT, the left gastric artery (LGA) was carefully checked and evaluated to determine whether it ran toward the liver, and if so, dissected. Additionally, a patch of the CT that merged into the abdominal aorta was reserved for future use.

Preparation of the CHA was the next step. The artery was skeletonized along the CT in the opposite direction to the liver. Then, the GDA was dissected free in the direction from the pancreas to the liver and checked thoroughly to determine whether the branches of the GDA arose in the liver. After the whole course of the GDA-CHA was dissected, the proper hepatic artery (PHA) was dissected from its direction toward the liver. Excess soft tissue around the porta hepatis was trimmed and ligated to avoid bleeding after revascularization of the PV. The dissection was stopped when the left and right hepatic arteries (RHAs) were clear of the PHA. After the right gastric artery was severed and sutured, the preparation of the hepatic artery was complete.

Next, the stump of the round ligament of the liver was ligated, and the incision of the gallbladder closed. A liver biopsy was usually taken from the left lobe. However, if it was a marginal donor liver, the biopsy was performed at the time of liver harvesting.

#### 2.4.5 Step 5: vascular leak checks

After back-table preparation, donor livers were weighed and re-perfused with organ preservation solution through the PV cannula. A PV leak check was undertaken first, followed by an IVC leak check. If a leak was detected, it was closed with sutures. In the case of classic liver transplantation, the upper and lower openings of the hepatic portion of the IVC were closed with a vascular clamp. For modified piggyback liver transplantation, the upper and lower hepatic IVC openings were sutured. Next, the hepatic artery was checked with an organ preservation solution from the CT or GDA for any leakage (Supplementary Video S2). Last, the cystic duct was clamped and the biliary tract was flushed through with organ preservation solution from the CBD. After back-table preparation, the donor livers were scrutinized again and stored at 0°C–4°C for future implantation.

#### 2.4.6 Other factors that influence post-reperfusion bleeding

Thrombus is managed through thrombectomy. If there was stenosis in the PV, the vessel was excised and the healthy stump was used for vascular anastomosis.

All shunts ≥1 cm were ligated to ensure portal venous flow. This step was typically performed after completing arterial anastomosis because a portal venous flow opening can significantly reduce the pressure in these shunts, thus minimizing the risk of significant bleeding during their handling. Ligation of the shunts improves portal venous flow without causing additional bleeding, as hemostasis has been checked twice before this step (before hepatic artery anastomosis and before bile duct anastomosis). Before concluding the surgery, we confirmed portal venous flow velocity to be above 20 cm/s using ultrasound.

### 2.5 Definition of a high-quality vs. low-quality hepatic artery preparation

**Figure 4:**
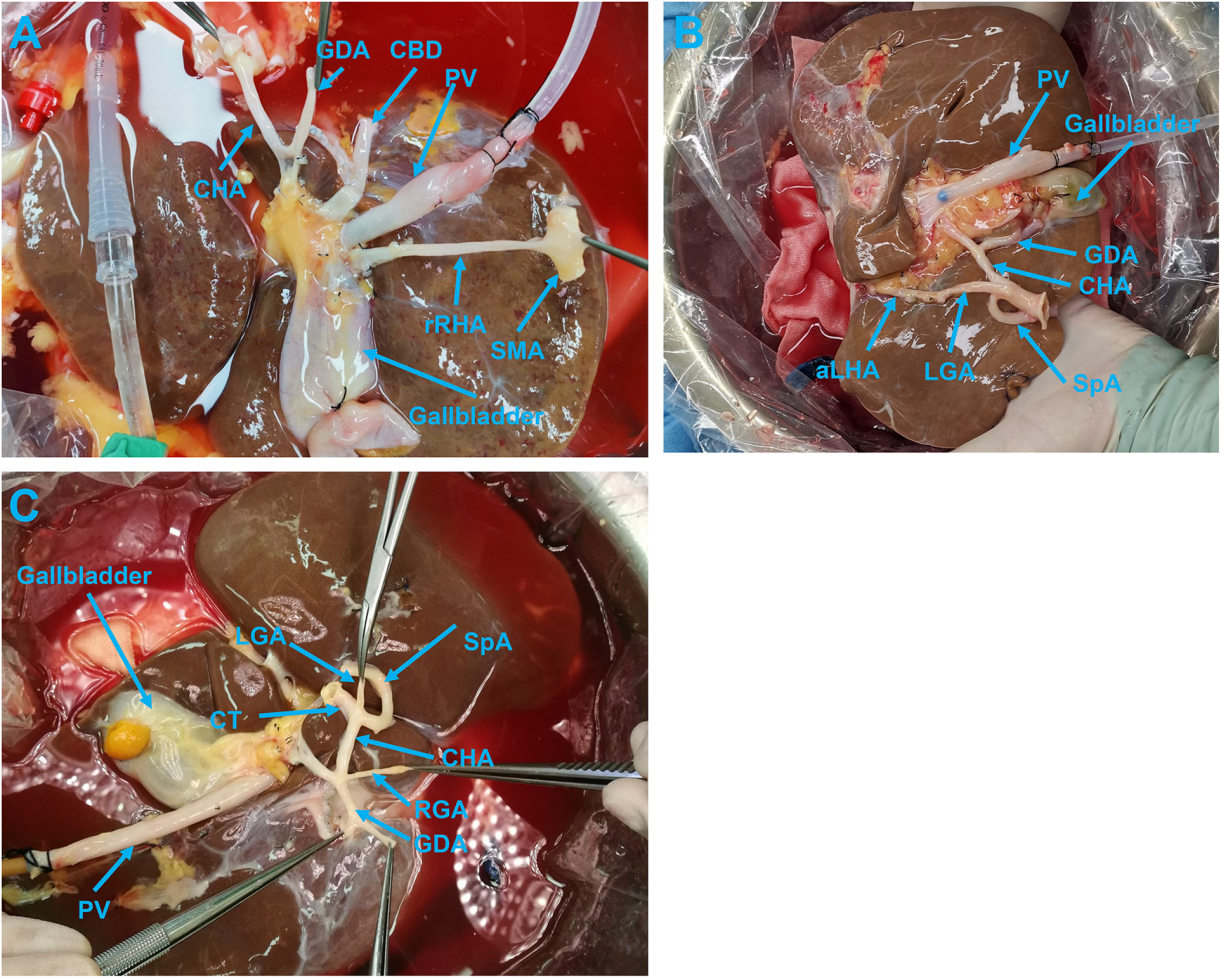
**(A)** A donor liver with high-quality preparation of aberrant hepatic artery. **(B)** Donor liver with an aberrant hepatic artery showing high-quality back-table preparation of arteries. **(C)** Donor liver without an aberrant hepatic artery showing high-quality back-table preparations of arteries. aLHA, accessory left hepatic artery; CBD, common bile duct; CHA, common hepatic artery; CT, celiac trunk; GDA, gastroduodenal artery; LGA, left gastric artery; PV, portal vein; RGA, right gastric artery; rRHA, replacement right hepatic artery; SMA, superior mesenteric artery; SpA, splenic artery.

For aberrant hepatic arteries originating from the SMA (Supplementary Figure S1A), high-quality preparation was defined as preservation of the entire aberrant hepatic artery, with a portion or patch of the SMA (Supplementary Video S3). However, if only the entire aberrant hepatic artery was retained without the SMA segment or patch, it was referred to as a low-quality preparation. Also, if the aberrant hepatic artery was severed within 3 cm of its liver entrance side, it was considered to be an injury (Figure 4A).

For the aberrant hepatic artery originated from the LGA (Supplementary Figure S1B), the preservation of the hepatic-LGA-CT, also known as the PHA-CHA-CT-LGA-aberrant hepatic artery loop, was accepted as a high-quality preparation (Supplementary Video S3). If the entire course of the aberrant hepatic artery-LGA was preserved but resected at its exit site from the CT, this preparation was considered to be a low-quality one. However, if the aberrant hepatic artery preparation did not go beyond 3 cm from the liver side, it was defined as an injury (Figure 4B).

In the preparation of hepatic arteries without variant structures, if the left and RHA-PHA-CHA-CT-abdominal aorta sleeve was preserved completely, and the lengths of the GDA, RGA, LGA, and SpA were long enough for subsequent trimming, the preparation was graded as high-quality trimming (Figure 4C).

### 2.6 Reconstruction of the IVC, PV, hepatic artery and bile duct

Reconstruction of the IVC in classic liver transplantation surgery involves end-to-end anastomosis at both ends of the IVC. In modified piggyback liver transplantation surgery, the IVC is connected using side-to-side anastomosis, employing 4-0 Prolene running sutures. PV reconstruction utilizes end-to-end anastomosis with 6-0 Prolene running sutures. Biliary ducts are connected via end-to-end anastomosis using 6-0 PDS running sutures or with running sutures on the posterior wall and interrupted sutures on the anterior wall. Arterial reconstruction employs 7-0 or 8-0 Prolene running sutures. In the absence of variations, end-to-end anastomosis is performed between the donor PHA/CHA and the recipient PHA/CHA. Variations in the RHA typically anastomose with the graft's GDA/SpA. Variations in the left hepatic artery (LHA) may anastomose with the graft's GDA/right gastric artery/SpA. Subsequently, the graft's CHA/abdominal aorta is anastomosed with the recipient's hepatic artery. When the diameter of the variant artery is ≤1 mm, or after CHA perfusion, if retrograde flushing was observed from the variant artery, confirming communication with the arterial trunk, reconstruction of the variant artery was deemed unnecessary.

### 2.7 Data collection and statistical analysis methods

The choice of treatment groups was determined by the patients and can be considered a random distribution. The following data were collected from operating notes, namely, graft weight, cold ischemia time, DLBTP time, the interval between the completion of donor liver preparation and its implantation, the variations of hepatic arteries, the number and proportion of high vs. low-quality back-table preparations, and injury to the hepatic artery. The time taken to control hemorrhage, the amount of blood, and bleeding sites due to a significant vascular break after opening the PV were also analyzed.

The transplant surgeons were interviewed about their surgical experiences with the procedural back-table preparation, and their subjective experiences were extracted from questionnaires of whether repairs to blood vessels or bile ducts hindered the vascular anastomosis of the IVC, PV, hepatic artery, or the bile duct and the degree of bleeding.

Data were analyzed using SPSS ver. 20 (IBM Corporation, Armonk, NY, USA). First, it had been confirmed whether each indicator followed a normal distribution. If indicators were continuous numerical values, analysis was conducted using the mean ± SD. Between-group comparisons were performed using Student's *t*-test for two groups and ANOVA for three groups to observe any inter-group differences in trends. When differences existed, between-group comparisons were performed by employing the Kruskal–Wallis test, and the mean ± SD was used for representation of the results. In cases where the distribution was not normal, between-group comparisons required the use of the Wilcoxon rank sum test to assess significant differences, represented by median and interquartile ranges. For categorical variables, the chi-squared (*χ*²) or Fisher's exact tests were used. A *P*-value of <0.05 was considered to be a statistically significant difference.

## 3 Results

### 3.1 Baseline demographic and clinical characteristics of patients

In total, 43 patients who underwent additional back-table procedures by two transplant surgeons, each having completed over 50 back-table procedures, were assigned to the control (Group A). A total of 160 patients who underwent the proceduralized DLBTP technique used in this study by another surgeon, who had completed 20 back-table procedures previously, were assigned to the experimental group (Group B). The majority of patients' primary condition was hepatocellular carcinoma, with 24 (55.8%) in Group A and 86 (53.8%) in Group B, while liver donors primarily came from individuals after brain death in both groups. For the situation of collateral vessels or varices, in Group A, there were four patients with large spontaneous portosystemic shunts, all of which were coronary veins. In Group B, there were 11 patients with large shunts, seven of which were coronary veins and four splenorenal shunts. There were no significant differences between the two groups for other clinical indicators such as portal venous thrombosis and collateral or varicose veins, as well as demographic baseline indicators (Table 1). Individual superficial tears of the liver capsule were managed during back-table surgery with a U-shaped suture or addressed post-implantation through electrocoagulation. Some patients had pericholedochal varices, which were relatively small and did not significantly interfere with surgical procedures. Neither group exhibited moderate or severe fatty liver, while all transplanted livers were devoid of significant traumatic injury.

**Table 1:** Baseline demographic and clinical characteristics of patients.

	Group A (*N* = 43)	Group B (*N* = 160)	*P*-value
Age, years			0.91
Median (range)	51 (28, 71)	51 (19, 72)	
Mean ± SD	50.35 ± 9.71	50.56 ± 10.98	
Gender—*n* (%)			0.51
Male	37 (86.0)	128 (80.0)	
Female	6 (14.0)	32 (20.0)	
Liver donor—*n* (%)			0.71
Donors after circulatory death (DCD)	11 (25.6)	47 (29.4)	
Donors after brain death (DBD)	32 (74.4)	113 (70.6)	
Primary disease—*n* (%)			0.21
Hepatocellular carcinoma	24 (55.8)	86 (53.8)	
Liver failure	7 (16.3)	14 (8.8)	
Liver cirrhosis	9 (20.9)	55 (34.4)	
Hepatic echinococcosis	2 (4.7)	2 (1.3)	
Caroli's disease	1 (2.3)	3 (1.9)	
Portal venous thrombosis—*n* (%)			0.84
Yerdal I	6 (14.0)	31 (19.4)	
Yerdel II	3 (7.0)	8 (5.0)	
Yerdel III	0	2 (1.3)	
Collateral or varicose veins—*n* (%)			0.37
Large spontaneous portosystemic shunts	4 (9.3)	11 (6.9)	
Coronary vein	4 (9.3)	7 (44.4)	
Splenorenal shunt	0	4 (2.5)	

### 3.2 Surgical outcomes in Group A

The operation-related results are listed as follows: a mean donor liver weight of 1,470 g (range, 1,030–1,950 g); a cold ischemia time of 6.8 h (range, 3–12.4 h); the interval time between the completion of donor liver preparation and liver implantation was 2.3 h (range, 0.7–8.6 h); and the back-table donor liver trimming time was 0.9 h (range, 0.7–1.2 h). The total number of hepatic artery variants was seven cases, including four variant LHAs from the LGA (Michel's Type II/V), two variant RHAs from the SMA (Michel's Type III/VI), and one variant LHA combined variant RHA from the LGA and SMA, respectively (Michel's Type IV/VII/VIII). Among 43 preparations, 39 hepatic arteries had high-quality back-table preparation, while 2 aberrant LHA and 2 aberrant RHA had low-quality preparations that accounted for 9.3% of the total cases and 57.1% of the cases with an aberrant hepatic artery (Table 2).

**Table 2:** Comparison of surgery outcomes.

	Group A (*N* = 43)	Group B (*N* = 160)	*P*-value
Liver weight, g			0.28
Median (range)	1,470 (1,030–1,950)	1,360 (890.0–2,030)	
Mean ± SD	1,509 ± 26	1,551 ± 256	
Cold ischemia time, h			
Median (range)	6.8 (3.0–12.4)	5.9 (3.7–10.6)	0.68
Mean ± SD	6.61 ± 1.85	6.48 ± 1.83	
Back-table donor liver trimming time, h			<0.001
Median (range)	0.9 (0.7–1.2)	1.4 (1.1–1.8)	
Mean ± SD	0.92 ± 0.17	1.37 ± 0.18	
Hepatic artery variants—*n* (%)	7 (16.3)	23 (14.4)	0.81
LHAs from the LGA (Michel's type II/V)	4 (9.3)	15 (9.4)	
RHAs from the SMA (Michel's type III/VI)	2 (4.7)	5 (3.1)	
LHA combined variant RHA from the LGA and SMA	1 (2.3)	2 (1.3)	
RHA from the GDA	0	1 (0.6)	
Back-table preparation—*n* (%)			0.04
High-quality	39 (90.7)	157 (98.1)	
Low-quality	4 (9.3)	3 (1.9)	
After PV unclamped			<0.001
Time to stop bleeding, h			
Median (range)	0.5 (0.25, 0.83)	0.3 (0.17, 0.58)	
Mean ± SD	0.52 ± 0.12	0.36 ± 0.13	
Blood loss, ml	180 (60, 320)	60 (30, 240)	<0.001
Median (range)	184 ± 69	79 ± 49	
Mean ± SD			
Location of major bleeding due to ruptured blood vessels—*n* (%)			
IVC	5 (11.6)	2 (1.3)	1.00
PV	2 (4.7)	0	

GDA, gastroduodenal artery; IVC, inferior vena cava; LGA, left gastric artery; LHA, left hepatic artery; PV, portal vein; RHA, right hepatic artery; SMA, superior mesenteric artery.

After the PV was unclamped, the time to stop bleeding was 0.5 h (range, 0.25–0.83 h), and the amount of blood loss was 180 ml (range, 60–320 ml). The locations of major bleeders due to vascular ruptures were IVC in five cases; PV in two cases; and the remaining main bleeding sites included the region where the adrenal glands adhered to the liver and the soft tissues of the hilar area. The trimming of blood vessels and bile ducts had no negative impact on the vascular anastomosis of the IVC, PV, hepatic artery, or bile duct. Twenty-six of these grafts were evaluated by two transplant surgeons, who commented that the soft tissue in the first porta hepatis area had excessive blood oozing after the blood flow was made patent (Table 2).

### 3.3 Surgical outcomes in Group B

A total of 160 patients was enrolled in Group B, with the mean weight of the graft being 1,360 g (range, 890–2,030 g), the cold ischemia time 5.9 h (range, 3.7–10.6 h), and the interval between completion of liver trimming and donor liver implantation 1.8 h (range, 0.3–6.3 h), all of which were found not to be significantly different from the control group values. Moreover, the DLBTP time was 1.4 h (range, 1.1–1.8 h), which was longer than that of the control group (*P* < 0.05). The total number of hepatic artery variations was 23 cases, including 15 cases of variant LHA from LGA (Michel's Type II/V), 5 cases of variant RHA of SMA (Michel's Type III/VI), 2 cases of variant LHA from LGA combined with variant RHA from the SMA (Michel's Type IV/VII/VIII), and 1 case of RHA from the GDA. Among the 160 cases, 157 had high-quality hepatic arteries, while only 2 cases of variant LHA were noted and 1 case of variant RHA underwent low-quality preparation, which represented 1.9% of the total number and 13.0% of the variances of the hepatic artery. The probability of low-quality preparation of the hepatic artery in the experimental group was significantly lower than in the control group (*P* < 0.05). The time for stemming bleeding and the amount of blood loss after the PV were opened was 0.3 h (range, 0.17–0.58 h) and 60 ml (range, 30–240 ml), respectively, in the experimental group, which were less than in the control group (*P* < 0.05). The sites of bleeding oozing, due to significant vascular breakage after opening the PV, were localized at the IVC rupture and the rest of the bleeding was around the soft tissue in the hilar region. Vascular and bile duct preparation had no negative effects on the vascular anastomosis of the IVC, PV, hepatic artery, or bile duct. Of 160 cases, 127 were evaluated by three transplant surgeons who gave their opinions compared with traditional back-table donor liver preparation and reported that the procedural preparation significantly reduced total bleeding after opening the PV. Also, there was less soft tissue in the hepatic portal region, which made it easier to implement the vascular anastomosis to prevent the implantation from exhibiting significant bleeding (Table 2).

### 3.4 Safety

After surgery, we usually give anticoagulants to patients to prevent arterial thrombosis. An early postoperative prophylactic dose of low molecular weight heparin was administered, transitioning to oral anticoagulants/antiplatelet drugs 1 week postoperatively, including rivaroxaban/aspirin.

In Group A, two patients had hepatic artery stenosis, with one patient experiencing HAT requiring reanastomosis surgery (considered related to SpA steal syndrome, with concurrent SpA ligation). The peak values of transaminases ALT were 878.77 ± 707.23 U/L, AST 2,270.51 ± 1,698.15 U/L, and delayed graft function occurred in two patients. Three patients with postoperative hepatic artery-related bile duct strictures underwent endoscopic retrograde cholangiopancreatography (ERCP) treatment, and two had postoperative bleeding that required reoperation for hemostasis.

In Group B, there were five hepatic artery stenosis (three were related to SpA theft and embolized the main SpA after intervention), but there were no cases of HAT, and the peak of transaminase ALT was 669.87 ± 703.71 U/L (*P* = 0.08). AST 1,563.66 ± 1,436.76 (*P* = 0.01), while delayed graft function occurred in three patients. After the operation, seven patients with hepatic artery-related biliary tract stenosis were treated with ERCP. Reoperation was performed to stop bleeding in three patients. There were no occurrences of aneurysm or primary non-function in either group (Table 3).

**Table 3:** Comparison of safety during surgery.

	Group A (*N* = 43)	Group B (*N* = 160)	*P*-value
Hepatic artery stenosis—*n* (%)	2 (4.6)	5 (3.1)	0.64
Hepatic artery thrombosis—*n* (%)	1 (2.3)	0	0.21
ALT (U/L), mean ± SD	878.77 ± 707.23	669.87 ± 703.71	0.08
AST (U/L), mean ± SD	2,270.51 ± 1,698.15	1,563.66 ± 1,436.76	0.01
Delayed graft function—*n* (%)	2 (4.6)	3 (1.9)	0.28
Hepatic artery-related bile duct stricture—*n* (%)	3 (7.0)	7 (4.4)	0.44
Reoperation to stop bleeding—*n* (%)	2 (4.6)	3 (1.9)	0.28
Aneurysm—*n* (%)	0	0	
Primary non-function—*n* (%)	0	0	

ALT, alanine aminotransferase; AST, aspartate aminotransferase.

## 4 Discussion

The aim of DLBTP is to enable surgeons to quickly and easily master surgery and reduce blood loss during liver transplant procedures through a modular and improved surgical method. This not only reduces the concern of the surgeon about bleeding during the operation, leading to a clearer and cleaner surgical field, but also, more importantly, reduces patient risk by minimizing blood loss. Moreover, this anastomotic technique, considering various vascular variations and preserving vessels effectively, facilitates subsequent vascular anastomosis, which allows liver transplant surgeons the flexibility to choose the most suitable vascular anastomosis method based on individual circumstances.

The surgical approach of the control group was not modularized and standardized. The PV was handled starting from the SMV toward the hepatic hilum, while the artery was dissected starting from the abdominal aorta along the vessel. This approach contrasts with conventional hepatobiliary pancreatic surgeries, such as surgery for bile duct cancer at the hepatic hilum and pancreaticoduodenectomy, where key liver vessels cannot be readily and directly located. Such a procedure involves certain differences in various back-table surgeries, especially when there are vascular variations. For example, in the presence of variant hepatic arteries, this approach cannot rapidly identify all arterial vessels entering the liver. In the surgical procedures of the experimental group, donor livers were re-perfused through the PV, hepatic artery, and bile duct, while in the control group, perfusion was usually only carried out through the PV.

Intraoperative hemorrhage is recognized as one of the major complications of OLT_X_, and there are various factors affecting bleeding during transplantation, including several that are not related to technical aspects of the operation. However, our focus was on assessing bleeding after liver graft implantation, specifically calculating the bleeding situation after liver reperfusion, in the hope of reducing bleeding at this stage of the operation. At this point, patients had already undergone thorough vascular anastomosis preparation with hemostasis, and additionally, there were analyses of preoperative conditions, coagulation function, and platelet numbers. Technically, surgical approaches seek to maximize the protection of potentially variable vessels, preserve a sufficient length of them for anastomosis, and minimize bleeding during liver implantation, thereby reducing the surgical risks during vascular anastomosis. With refinements on the operation of donor liver replantation, the prevention of intraoperative bleeding has been shifted to emphasize DLBTP, which routinely aims to trim the connective tissue around the porta hepatis to improve the quality of major vascular anastomosis in this region, and to skeletonize carefully the hepatic arteries to identify aberrant vessels to avoid the possibility of hepatic arterial thrombosis (4).

### 4.1 Procedural back-table preparation of the hepatic artery

Surgically, hepatic arterial variations have been of considerable ill consequence in liver transplants, during which inadvertent or iatrogenic injuries frequently occur in the aberrant vasculature, various biliary tract complications, liver dysfunction, and even loss of grafts (6).

Following Michel's classification, the most frequent variation of the CHA is a branch out of the SMA, with the LHA derived from the LGA and the RHA from the SMA (7). Additionally, the LHAs and RHAs rarely arise from the abdominal aorta, CT, SpA, or GDA (1).

To avoid damaging the grafted hepatic artery in OLT_X_, the aberrant hepatic artery should first be identified and protected intact during the preparation of the donor liver. During the back-table extracorporeal preparation of the donor liver, surgeons should recognize all the variations of the hepatic artery and especially focus on distinguishing the RHA at the ventral/dorsal side of the PV and the aberrant LHA originating from the LGA in the hepatogastric ligament (8, 9).

Moreover, in comparison with conventional donor liver preparation, the back-table donor liver preparation procedure emphasizes separating the PV and bile duct from the hepatoduodenal ligament, which subsequently leaves the hepatic artery as the only structure either in the hepatoduodenal or hepatogastric ligaments. Another unique feature of the procedure is that the dissection started at the upper margin of the pancreas, which is far from the hepatic hilar. Since the diameter of the blood vessel at this level is relatively large, the possibility of injury is low, and even if damage occurs, the hepatic artery can be easily repaired. Therefore, it was not necessary to repair the hepatic artery routed from the abdominal aorta.

In the process of back-table trimming, the posterior wall of the PV, if the RHA originating from the SMA was visualized, the SMA segment was trimmed and preserved for vascular reconstruction. If the LHA originating from the LGA was found during the processing of the hepatogastric ligament, the entire left gastric LHA was retained. Although overlong aberrant right and LHAs usually needed to be further trimmed for vascular reconstruction and anastomoses, it was still necessary to maintain blood vessels of sufficient length together with their originating vasculature.

For an aberrant hepatic artery with a smaller diameter (<2 mm), its communication with the PHA in the liver can be assessed by pressure infusion. If fluid gushes out from this type of aberrant hepatic artery during the infusion, the hepatic artery can be safely ligated. In the present study, 11 cases of left aberrant hepatic artery originating from the LGA and 1 case of right aberrant hepatic artery originating from SMA displayed ≤1 mm diameters and were completely ligated, suggesting that the left aberrant hepatic artery branching from the LGA was prone to have a smaller diameter and could be sacrificed without any clinical consequence. However, for the variant hepatic artery with a diameter >2 mm, preservation for further vascular reconstruction was necessary.

Before this article, as far as we are aware, no study has been designated to either discuss a procedural back-table preparation of donor liver or to illustrate how precisely to identify the hepatic artery variations in donor livers. Most of the previous reports assumed that the arterial inflow conduit should be dissected from the aortic patch to the bifurcation of the GDA (10, 11). However, the arteries should be tracked to detect aberrant hepatic artery variations. Also, the risk of vascular injury is significantly higher when the hepatic artery is procured from larger diameter portions than from the smaller diameter arteries. Anatomically, arteries entering the liver must pass through the hepatoduodenal ligament or the hepatogastric ligament. Thus, the careful dissection of these two regions can directly identify the arterial tunnel of the liver. In practice, the CHA could be easily found by dissecting through the space between the eight lymph node groups and the upper edge of the pancreas. Then, the artery could be trimmed on both sides to complete its preparation.

**Figure 5:**
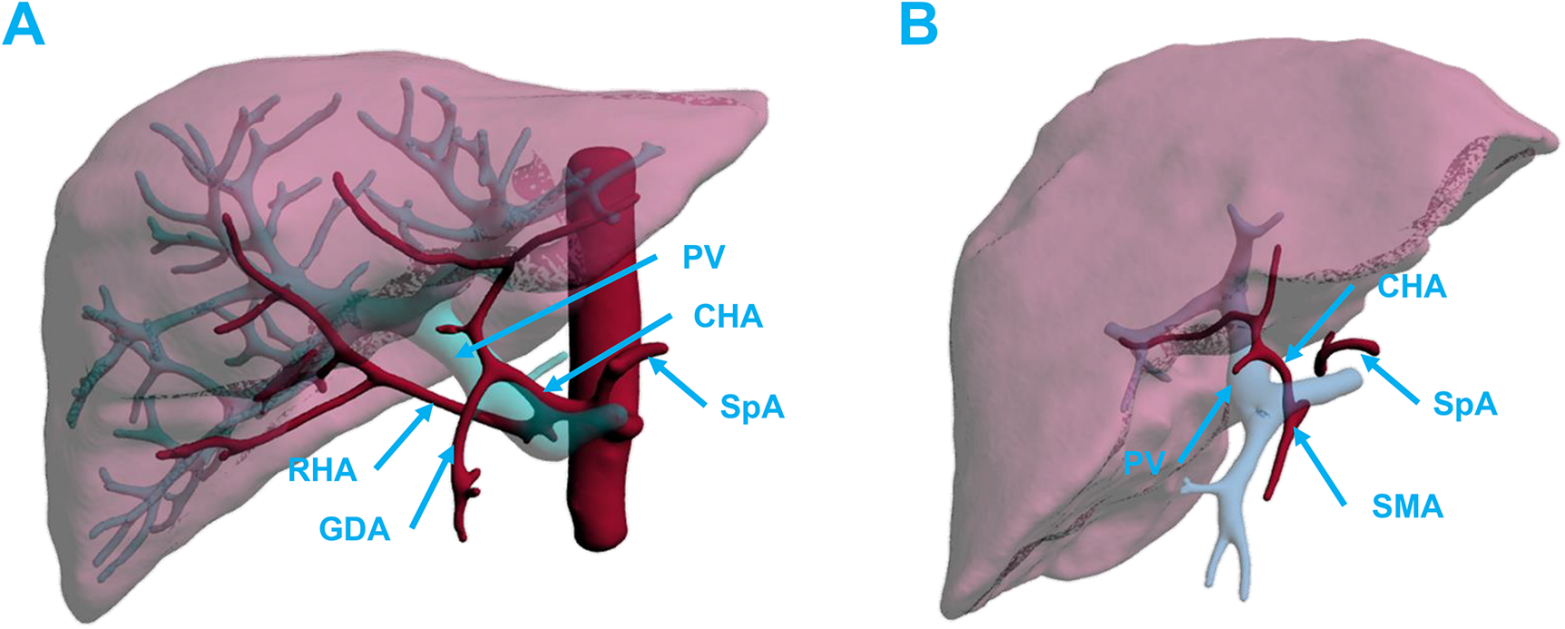
**(A)** An aberrant RHA arising from the common hepatic artery. **(B)** Aberrant CHA arising from the SMA that did not run across the dorsal PV. CBD, common bile duct; CHA, common hepatic artery; GDA, gastroduodenal artery; LGA, left gastric artery; PV, portal vein; RHA, right hepatic artery; SMA, superior mesenteric artery; SpA, splenic artery.

The procedure mentioned in this article of actively retaining the SMA to the right side of the liver is suitable for most right aberrant hepatic arteries that originate from the SMA. However, in rare cases, when the hepatic artery that originated from the SMA did not circumvent the dorsal side of the PV, but instead entered the liver from the left side of the PV, there was an increased risk of arterial injury. Special care should be taken when dissecting the non-SMA origin hepatic arterial variant (such as the variant RHA originating either from the CHA, CT, or SpA) that runs behind the PV. It should not be blindly assumed that the vessel originated from the SMA and could be cut; otherwise, vascular damage may well occur (Figure 5).

### 4.2 Back-table extracorporeal preparation of the PV and IVC of the donor liver

The main trunks of the PV s are usually devoid of large branches about 4–5 cm from the hepatic portal, and the first 2–3 branches from the main trunks were routinely suture-ligated to avoid completely the possibility of unnecessary bleeding from the shedding of the knots and to create a sufficient length of the portal SMV for future vascular anastomosis. To scrutinize the leakage of the PV, the vessel wall was closely inspected for small breaks, which may have affected perfusion of the implanted liver or caused significant bleeding.

Previous studies have shown that the major bleeding areas of the vena cava were mainly located in the posterior wall, suggesting the existence of branches to the posterior abdominal wall and phrenic vein. Furthermore, a vascular break in the IVC can be detected using the side leakage method (6). However, we believe that testing with PV perfusion is a more physiological examination and can also be very effective in locating any breaks along related vessels.

### 4.3 Back-table extracorporeal preparation of soft tissue, lymph node, and bile duct around the donor liver

During back-table donor liver preparation, excess soft tissue around the porta hepatis was carefully removed to optimize the operation for the vascular and biliary ductal anastomosis. Since the tissue was ligated during the trimming process, this may help reduce the chances of the development of a lymphatic fistula and ascites. Furthermore, when trimming the biliary ducts, the dissection should not be too close to or run too far long along the ducts to safeguard a sufficient blood supply to the ducts. This fact was evidenced by observing excellent blood flow at the end of the bile duct before the ductal anastomosis was constructed.

### 4.4 Potential implication for clinical practice

Despite variations in techniques across different centers, our goal was to identify a relatively standardized and procedural approach to make the surgical process more accessible and operationally convenient, while maximizing the protection of potentially variable vessels, preserving a sufficient length of vessels for anastomosis, and minimizing bleeding during liver implantation, thereby reducing risks and challenges that arise during vascular anastomosis. During the open circulation of the transplanted organ, challenges arose in terms of anesthesia and monitoring the patient's vital signs. Therefore, precise and procedural operations made this process more controllable.

### 4.5 Limitations

Apart from the retrospective design of the study, there were some confounding factors such as additional information about donors and recipients. Also, the limited sample size introduced certain constraints. Investigating a larger cohort of patients and conducting more comparative studies with a randomized control group will be required to validate further the practicality of the newly introduced method.

## 5 Conclusions

Our procedural donor liver extracorporeal preparation was a time-consuming procedure because we treated it as an intracorporeal operation with excessive ligations and suture ligations. However, we experienced significant reductions in the amount of bleeding and the time for controlling bleeding once the PV was unclamped after the donor liver was transplanted. Moreover, this back-table donor liver preparation procedure made the aberrant hepatic arteries easier to identify during artery skeletonization thus avoiding damage to the artery and minimizing the chance of postoperative HAT. Taken together, the proceduralized back-table donor liver preparation described enriched the first-hand surgical experience for our transplant surgeons. However, whether the procedure can improve the prognosis of liver transplant patients will require further research.

## Data Availability

The raw data supporting the conclusions of this article will be made available by the authors, without undue reservation.

## Supplementary material

Supplementary Video S1 The back-table donor liver preparation procedure.

Supplementary Video S2 Step 5: vascular leak checks.

Supplementary Video S3 Definition of high-quality hepatic artery preparation.

Supplementary Figure S1 Common hepatic artery variants. **(A)** rRHA from the SMA. **(B)** aLHA from LGA. aLHA, accessory left hepatic artery; LGA, left gastric artery; rRHA, replaced right hepatic artery; SMA, superior mesenteric artery.

## References

[1] Rather SA Nayeem MA Agarwal S Goyal N Gupta S. Vascular complications in living donor liver transplantation at a high-volume center: evolving protocols and trends observed over 10 years. Liver Transpl*.* (2017) 23(4):457–64. 10.1002/lt.24682 27880991

[2] Yoon YI Lee SG Moon DB Park GC Ahn CS Cho YP Microsurgical hepatic artery reconstruction in deceased donor liver transplantation for reduced arterial complications. Transplant Proc*.* (2021) 53(5):1645–52. 10.1016/j.transproceed.2021.04.009 34001348

[3] Pan ET Yoeli D Galvan NTN Kueht ML Cotton RT O’Mahony CA Cold ischemia time is an important risk factor for post–liver transplant prolonged length of stay. Liver Transpl*.* (2018) 24(6):762–8. 10.1002/lt.25040 29476693

[4] Hejazi Kenari SK Zimmerman A Eslami M Saidi RF. Current state of art management for vascular complications after liver transplantation. Middle East J Dig Dis*.* (2014) 6(3):121–30. 25093059 PMC4119668

[5] Tan-Tam C Segedi M Buczkowski A Hussaini T Yoshida EM Chung S Surgical complications of liver transplantation. AME Med J. (2018) 3:107. 10.21037/amj.2018.10.02

[6] Piardi T Lhuaire M Bruno O Memeo R Pessaux P Kianmanesh R Vascular complications following liver transplantation: a literature review of advances in 2015. World J Hepatol*.* (2016) 8(1):36–57. 10.4254/wjh.v8.i1.36 26783420 PMC4705452

[7] Noussios G Dimitriou I Chatzis I Katsourakis A. The main anatomic variations of the hepatic artery and their importance in surgical practice: review of the literature. J Clin Med Res*.* (2017) 9(4):248–52. 10.14740/jocmr2902w 28270883 PMC5330766

[8] Suzuki T Nakayasu A Kawabe K Takeda H Honjo I. Surgical significance of anatomic variations of the hepatic artery. Am J Surg. (1971) 122(4):505–12. 10.1016/0002-9610(71)90476-4 5098656

[9] Perez-Saborido B Pacheco-Sanchez D Barrera-Rebollo A Asensio-Diaz E Pinto-Fuentes P Sarmentero-Prieto JC Incidence, management, and results of vascular complications after liver transplantation. Transplant Proc*.* (2011) 43(3):749–50. 10.1016/j.transproceed.2011.01.104 21486590

[10] Karakoyun R Romano A Yao M Dlugosz R Ericzon B-G Nowak G. Impact of hepatic artery variations and reconstructions on the outcome of orthotopic liver transplantation. World J Surg*.* (2020) 44(6):1954–65. 10.1007/s00268-020-05406-4 32030440

[11] Lin T-S Vishnu Prasad NR Chen C-L Yang JC-S Chiang Y-C Kuo P-J What happened in 133 consecutive hepatic artery reconstruction in liver transplantation in 1 year? Hepatobiliary Surg Nutr*.* (2019) 8(1):10–8. 10.21037/hbsn.2018.11.13 30881961 PMC6383017

