# Supplementary figures and images for: A novel proceduralized donor liver back-table preparation technique minimizes hemorrhage following liver implantation in orthotropic liver transplantation
Source: Front Surg. 2024 Dec 12;11:1356142. doi: 10.3389/fsurg.2024.1356142 (PMC11669602; doi:10.3389/fsurg.2024.1356142)

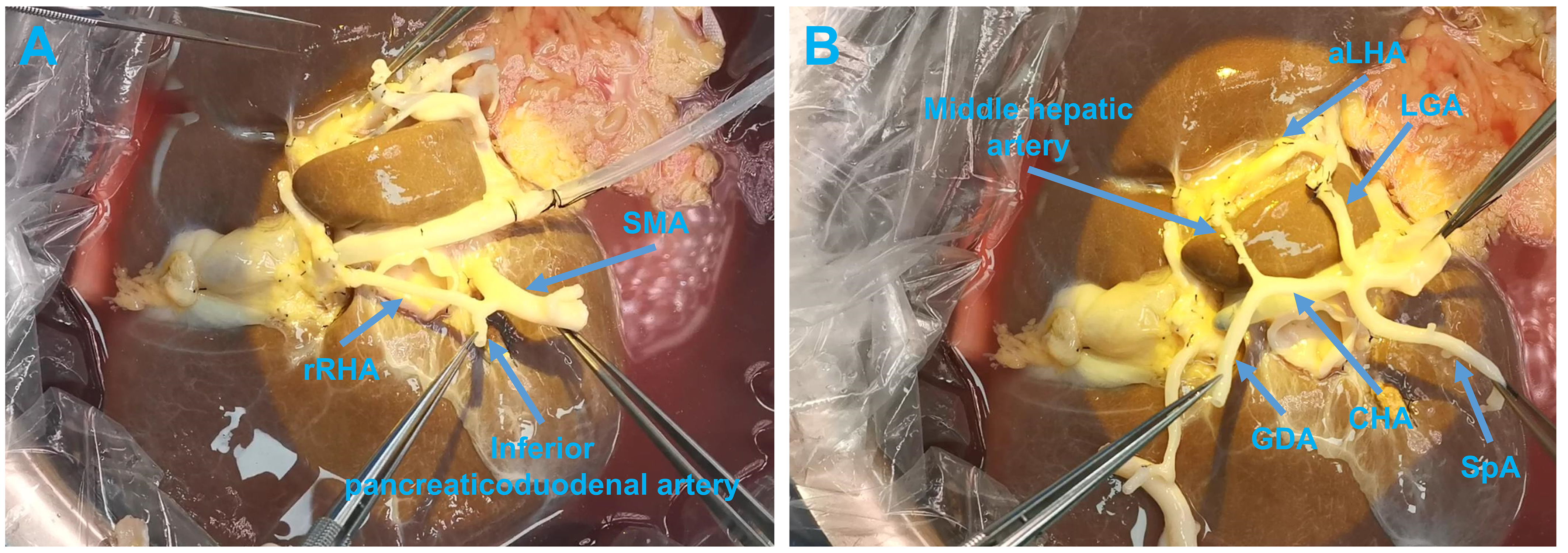

Supplement: Supplementary Figure S1 [file Image1.tif]
